# A Protein Diet Score, Including Plant and Animal Protein, Investigating the Association with HbA1c and eGFR—The PREVIEW Project

**DOI:** 10.3390/nu9070763

**Published:** 2017-07-17

**Authors:** Grith Møller, Diewertje Sluik, Christian Ritz, Vera Mikkilä, Olli T. Raitakari, Nina Hutri-Kähönen, Lars O. Dragsted, Thomas M. Larsen, Sally D. Poppitt, Marta P. Silvestre, Edith J.M. Feskens, Jennie Brand-Miller, Anne Raben

**Affiliations:** 1 Department of Nutrition, Exercise and Sports, Faculty of Science, University of Copenhagen, Rolighedsvej 26, 1958 Frederiksberg C, Denmark; ritz@nexs.ku.dk (C.R.); ldra@nexs.ku.dk (L.O.D.); tml@nexs.ku.dk (T.M.L.); ara@nexs.ku.dk (A.R.); 2 Division of Human Nutrition, Wageningen University, Stippeneng 4, 6708 WE Wageningen, The Netherlands; Diewertje.sluik@wur.nl (D.S.); edith.feskens@wur.nl (E.J.M.F.); 3 Department of Food and Environmental Sciences, University of Helsinki, 00014 Helsinki, Finland; vera.mikkila@aka.fi; 4 Research Centre of Applied and Preventive Cardiovascular Medicine, Kiinamyllynkatu 10, University of Turku, 20520 Turku, Finland; olli.raitakari@uta.fi; 5 Department of Clinical Physiology and Nuclear Medicine, Turku University Hospital, 20521 Turku, Finland; 6 Department of Pediatrics, Tampere University Hospital and Faculty of Medicine and Life Sciences, University of Tampere, 33014 Tampere, Finland; nina.hutri-kahonen@uta.fi; 7 Human Nutrition Unit, School of Biological Sciences, 18 Carrick Place, Mt Eden, University of Auckland, Auckland 1024, New Zealand; s.poppitt@auckland.ac.nz (S.D.P.); m.silvestre@auckland.ac.nz (M.P.S.); 8 School of Life and Environmental Sciences & Charles Perkins Centre, University of Sydney, Camperdown, NSW 2006, Australia; jennie.brandmiller@sydney.edu.au

**Keywords:** protein diet score, HbA1c, eGFR, healthy subjects, population studies

## Abstract

Higher-protein diets have been advocated for body-weight regulation for the past few decades. However, the potential health risks of these diets are still uncertain. We aimed to develop a protein score based on the quantity and source of protein, and to examine the association of the score with glycated haemoglobin (HbA1c) and estimated glomerular filtration rate (eGFR). Analyses were based on three population studies included in the PREVIEW project (PREVention of diabetes through lifestyle Intervention and population studies in Europe and around the World): NQplus, Lifelines, and the Young Finns Study. Cross-sectional data from food-frequency questionnaires (*n* = 76,777 subjects) were used to develop a protein score consisting of two components: 1) percentage of energy from total protein, and 2) plant to animal protein ratio. An inverse association between protein score and HbA1c (slope −0.02 ± 0.01 mmol/mol, *p* < 0.001) was seen in Lifelines. We found a positive association between the protein score and eGFR in Lifelines (slope 0.17 ± 0.02 mL/min/1.73 m^2^, *p* < 0.0001). Protein scoring might be a useful tool to assess both the effect of quantity and source of protein on health parameters. Further studies are needed to validate this newly developed protein score.

## 1. Introduction

A diet rich in protein, ranging from 1.2 to 1.6 g protein/kg/day, may improve body weight regulation [1,2,3]. Protein-rich diets also appear to supplement other strategies, such as energy restriction and physical activity, to combat the global obesity epidemic [4]. As obesity is an independent risk factor for type 2 diabetes (T2D), higher protein diets for weight regulation and maintenance might also be beneficial for the prevention of T2D [5]. Although the total amount of protein may be important, the source of protein will influence other components of the diet, such as dietary fibre and micronutrients. Therefore, protein source is likely to be an important determinant for health outcomes [6]. Dietary guidelines suggest moving towards a more plant-based diet [7]. Plant-derived diets provide a number of phytochemicals that have been associated with protection against many chronic diseases, but conversely compared to dietary proteins from animal sources, plant proteins lack sufficient amounts of key essential amino acids. In most industrialised countries, animal protein is the main source of dietary protein rather than plant protein, and is therefore often a differentially increased choice when total protein intake is increased. However, the consumption of animal products, especially red and processed meat, has been associated with an increased risk of diseases such as cancer, T2D and cardiovascular diseases [8,9,10]. The optimal plant to animal protein ratio in the diet has not yet been established. 

Firstly, we considered whether a higher protein intake might be associated with lower glycated haemoglobin (HbA1c). HbA1c levels measure average blood glucose concentration over the previous six to eight weeks; high concentrations are a risk factor for T2D. A recent meta-analysis of 32 randomised controlled trials showed a long-term positive effect of higher-protein diets on body weight management, which in turn could lead to lower HbA1c [11]. Several dietary intervention studies have also shown that higher protein diets can lower HbA1c directly, at least among T2D patients [12]. Secondly, we considered whether a higher protein intake may be associated with elevated estimated glomerular filtration rate (eGFR), an adverse indicator of renal function, since carefully controlled dietary studies show that higher protein diets may increase this marker of kidney function [13]. The current concern for adverse renal effects of higher protein diets derives from the detrimental effects of induced glomerular hyperfiltration [14]. Thirdly, with respect to protein quality, there are several lines of epidemiological evidence indicating that an increased consumption of plant protein may be associated with a reduced risk of cardiovascular disease, T2D [5,15] and inflammation [16], which might be ascribed in part to lower levels of HbA1c.

In the past three decades, studies have clearly shown that the relationship between dietary intake and health is very complex, with many interactions [17]. For all these reasons, examining composite indices of food and nutrient intake can be useful. Recently, a low-carbohydrate diet score was developed by Halton et al. [18] to classify women in the Nurses’ Health Study according to their relative levels of fat, protein—including animal and vegetable protein—and carbohydrate intake. This score was used to prospectively examine the association with the risk of coronary heart disease in this cohort.

A scoring tool to specifically assess quantity, as well as the source, of protein intake has to our knowledge not previously been developed. Therefore, we aimed to develop a protein diet score based on dietary protein quantity and source—plant or animal—as a tool to investigate the role of protein in T2D-related adverse metabolic health. We hypothesised that a protein score with a higher protein energy percentage (*E*%) within the acceptable macronutrient distribution range for protein [19], in combination with a higher plant to animal protein ratio, would be associated with a lower HbA1c level and possibly also an increase in eGFR.

## 2. Materials and Methods

### 2.1. Study Design and Population

This study included cross-sectional data from two Dutch and one Finnish observational studies, NQplus, Lifelines and The Young Finns Study, all part of the PREVIEW project (PREVention of diabetes through lifestyle Intervention and population studies in Europe and around the World) www.previewstudy.com, The overall aim of PREVIEW is to investigate the impact of a higher-protein, low glycemic index (GI) diet and physical activity regime for the prevention of T2D in overweight and obese adults and children at high risk of developing this disease [20].

The Nutrition Questionnaires plus study (the NQplus study) is a longitudinal observational study in Dutch adults within the surroundings of Wageningen, the Netherlands. Its main aims are to develop a national dietary assessment reference database for the future development and improvement of dietary assessment methods, to validate newly developed food frequency questionnaires (FFQs), and to study dietary factors and intermediate health outcomes. A total of 2048 individuals aged from 20–70 years took part in the study [21,22].

Lifelines is a large observational population-based cohort study of both adults and children conducted in the north of the Netherlands [23]. The overall aim of this study was to gain insight into the etiology of healthy aging in the general population. Lifelines was initiated in 2006 and baseline data have been collected for 167,729 individuals, aged 6 months–93 years. Regular follow-up measurements are planned.

The Young Finns Study is a multi-centre follow-up study in Finland. Its main goals are to determine the contribution made by childhood lifestyle, biological, and psychological measures to the risk of cardiovascular diseases in adulthood. At baseline in 1980, 3596 subjects aged 3–18 years were included, and the same subjects have now been followed for more than 30 years [23]. For the purposes of this cross-sectional investigation, we used measurements from the 2007 follow-up, when subjects were 30–45 years of age.

All subjects at baseline who had missing data on the FFQ, or subjects who had implausibly high (>3500 kcal) or low (<500 kcal) daily energy intake on the FFQ, were excluded [18]. Furthermore, subjects with missing values in either exposure or outcome variables were omitted. Also, subjects with a history of diabetes, hypertension, hypercholesterolemia, cardiovascular disease, cancer or kidney disease were excluded, because these diseases can cause alterations in the habitual diet. After these exclusions, a total of 76,777 subjects from Lifelines (*n* = 75,131), NQplus (*n* = 492), and The Young Finns Study (*n* = 1154) remained in the current analysis.

### 2.2. Assessment of Diet

NQplus used a validated 180 item semi-quantitative general FFQ to assess usual dietary intake [24,25]. Answer categories for frequency questions ranged between never per month to 6–7 days per week, and portion sizes were estimated using typical portion size estimates (e.g., glass, slice) and commonly used household measures. Average daily nutrient intakes were calculated by multiplying the frequency of consumption by portion size and nutrient content per gram using the Dutch food composition table of 2011 [26]. With respect to the definition of animal and vegetable protein, the Dutch food composition table distinguishes between these sources and was used for both NQplus and Lifelines.

In Lifelines, a newly developed FFQ consisting of 110 items was used to estimate intake of energy, fat, carbohydrate, protein and alcohol. Responses to food frequency questions ranged between never per month to 6–7 days per week, and portion sizes were estimated using typical portion size estimates and commonly used household measures. Average daily nutrient intakes were calculated by multiplying frequency of consumption by portion size and nutrient content per gram using the Dutch food composition table of 2011 [26].

In the Young Finns Study, a validated 131-item quantitative FFQ was used [27]. The food items were presented in 12 subgroups, e.g., dairy products, vegetables, or fruits and berries. After each subgroup, there were empty lines for subjects to add foods not listed in the questionnaire. The portion sizes were fixed and, if possible, specified using typical portion size estimates. The nine frequency categories ranged from never or seldom to six or more times a day. The food consumption and nutrient intakes were calculated by multiplying the frequency of food consumption by fixed portion sizes to obtain the weight of each listed food item consumed as an average per day [28]. The Finnish food composition database of 2008 was used for the nutrient calculations [29]. The database does not distinguish between animal or plant protein as separate nutrients; therefore, all foods were first classified into either “animal” or “plant” foods. Protein intakes were calculated by the source using these classes.

### 2.3. Calculation of the Protein Score

Following the methodology of Halton et al. [18], a protein score was developed based on relative cut-off points i.e., the population distribution. The scoring was set according to the hypothesis that a higher protein intake, as well as a higher plant to animal protein ratio, would be associated with improved markers of health outcomes, including a lower HbA1c (a measure of average blood glucose concentrations in the previous 6–8 weeks) and higher eGFR (a measure of good kidney function). Each study population was divided into 11 strata according to total protein intake (expressed as *E*%), and 11 strata according to plant to animal protein ratio. Subjects in the highest stratum of protein intake received 10 points, subjects in the next stratum received 9 points, and so on, down to subjects in the lowest stratum, who received 0 points. In terms of calculating plant to animal protein ratio, those with the highest intake of plant to animal protein received 10 points, and those with the lowest plant to animal protein ratio received 0 points. The sum of the points of each of the two components created the overall protein diet score, which ranged from 0 to 20 points. Therefore, a higher score reflects a higher energy percentage of total protein, and a higher plant to animal protein ratio, while a lower score reflects a lower protein and a lower plant to animal protein ratio. Each component of the score was also considered separately.

### 2.4. Validation of the Protein Score in NQplus Using a Urinary Biomarker

Nitrogen (N) from 24-h urine was determined in NQplus by the Kjeldahl technique (Foss KjeltecTM 2300 analyser, Foss Analytical, Hilleroed, Denmark). This allowed us to evaluate the validity of the protein *E*% component score based on FFQ versus nitrogen excretion in urine, assuming N balance. Adjustments for incomplete urine samples were also done by para-aminobenzoic acid [30].

An attenuation factor, usually between 0 and 1, was defined as the correlation between the self-reported N intakes using the FFQ and the measured N losses (assumed to be “true” intake) using the urinary biomarker [30,31].

### 2.5. Risk Factor Assessment

In NQplus, fasting venous blood was collected, and immediately centrifuged and stored at −80 °C until further analyses. Serum creatinine was determined using enzymatic methods via a Dimension Vista 1500 automated analyser or Roche Modular P800 chemistry analyser (Roche, Basel Switzerland). HbA1c was determined with a high-performance liquid chromatographic (HPLC) measurement technology using an ADAMSTM A1c HA-8160 analyser (A. Menarini Diagnostics, Florence, Italy). eGFR was estimated using the chronic kidney disease epidemiology collaboration creatinine equation (CKD-EPI) [32]. 

In Lifelines, fasting blood samples were processed on the day of collection and stored at −80 °C until analysis. Serum creatinine was measured on a Roche Modular P chemistry analyser (Grenzacherstrasse, Switzerland). The HbA1c level was measured using a turbidimetric inhibition immunoassay (COBAS INTEGRA 800 CTS analyser; Roche Diagnostics, Almere, the Netherlands), but standardised against the reference method of the International Federation of Clinical Chemistry and Laboratory Medicine (IFCC). eGFR was estimated using the chronic kidney disease epidemiology collaboration creatinine equation (CKD-EPI) [32].

In the Young Finns Study, fasting blood samples were collected and stored at −70 °C until analysis. Serum creatinine was determined photometrically (Creatinine reagent, Dublin, Ireland) on an AU400 analyser (Olympus, Tokyo, Japan). eGFR was estimated using the Modification of Diet in Renal Disease (MDRD) formula [33]. The HbA1c fraction in blood was measured by an ARCHITECT ci8200 analyser (Abbott Laboratories, Abbott Park, IL, USA). The concentration of HbA1c was measured immunoturbidimetrically with the microparticle agglutination inhibition method (HbA1c reagent; Thermo Fisher Scientific, Waltham, MA, USA).

### 2.6. Statistical Analyses

All statistical analyses were performed using *R* version 3.2.0 [34], and IBM SPSS Statistics 22. Separate but similar analyses as detailed below were carried out for each of the three studies. Associations between total protein score, HbA1c and eGFR were evaluated by means of analysis of covariance (ANCOVA). Furthermore, associations between the single components of the protein score (plant to animal protein ratio, animal protein (*E*%), plant protein (*E*%), and total protein intake (*E*%)) and HbA1c and eGFR were investigated using ANCOVA. The protein score and its components were adjusted for total energy intake by means of the residual method [35] before entering the analyses. Both unadjusted and adjusted analyses of associations between total protein score, HbA1c and eGFR were performed. The adjustment for possible confounders included: age (years), gender (male/female), education (low, medium and high), alcohol (0 g/day, 0–6 g/day, 6–12 g/day, ≥12 g/day), smoking status (never, former, current <10 cigarettes/day, current ≥10 cigarettes/day) and exercise. Low education meant no education or primary education; medium education meant lower or preparatory vocational education, lower general secondary education, intermediate vocational education or apprenticeship, higher general secondary education, or pre-university secondary education; high education meant higher vocational education or university). Smoking status was included as a confounding factor, because smoking has been associated with increased T2D risk [36] and may be an independent risk factor in the progression of kidney disease [37]. Light intense, moderate intense, and intense physical activity were measured in the metabolic equivalent of the task in minutes per week. 

Potential effect modification was assessed through stratified analyses for age, gender, and BMI categories. A *p*-value < 0.05 was considered statistically significant.

## 3. Results

### 3.1. Characteristics of the Study Populations

The average protein diet score ranged from 8.0 in the lower quartile to 12.0 in the upper quartile in NQplus, Lifelines and the Young Finns Study (Table 1). In NQplus, Lifelines and the Young Finns Study, 50% of the subjects had a protein score between 8.0–12.0. The mean daily protein intake ranges were 13.1–15.9 *E*% in NQplus, 13.3–16.0 *E*% in Lifelines, and 16.0–19.0 *E*% in the Young Finns Study. The median age of 39.0 years in the Young Finns Study was lower compared to NQplus (53.0 years) and Lifelines (44.0 years). In the Young Finns Study, median physical activity was 792 MET min/week, as compared with NQplus (2376 MET min/week) and Lifelines (2205 MET min/week). Furthermore, the median daily intake of animal protein was larger in the Young Finns Study (12.3 *E*%), as compared with NQplus (7.7 *E*%) and Lifelines (8.4 *E*%). In addition, the median intake of cereals was higher in both NQplus (190 g/day) and Lifelines (181 g/day) than in the Young Finns Study (124 g/day) (Table 1). 

In NQplus, we evaluated the validity of the protein score against nitrogen excretion in urine, after adjusting for incomplete urine samples. The attenuation factor for the protein score was 0.48, indicating a 48% average concordance between self-reported protein intake and the urine biomarker of protein intake.

### 3.2. Association between Protein Diet Score and HbA1c

There were no associations between the protein diet score and HbA1c in either NQplus, Lifelines or the Young Finns Study. After adjustments, an inverse association between the protein diet score and HbA1c (slope −0.02 ± 0.01 mmol/mol, *p* < 0.001, Table 2) was seen in Lifelines.

### 3.3. Association between Total E% and HbA1c

There was a positive association between total protein (*E*%) and HbA1c in Lifelines (slope 0.07 ± 0.01 mmol/mol, *p* < 0.001, Table 2). After adjustments, we found an inverse association between the total protein intake (*E*%) and HbA1c in Lifelines (slope −0.03 ± 0.01 *E*%/mmol/mol, *p* < 0.001, Table 2). In NQplus and the Young Finns Study, no associations were found, neither in the basic model nor after adjustments.

### 3.4. Association of Plant to Animal Protein Ratio and HbA1c

In Lifelines, we found an inverse association (slope −0.19 ± 0.04 mmol/mol, *p* < 0.001, Table 2) between the plant to animal protein ratio and HbA1c in the unadjusted model. After adjustments, this association did not remain.

### 3.5. Association between Protein Diet Score and eGFR

For eGFR, we found a positive association between the protein diet score and eGFR in NQplus (slope 0.53 ± 0.21 mL/min/1.73 m^2^, *p* = 0.02, Table 2), and an inverse association between the protein diet score and eGFR in Lifelines (slope −0.08 ± 0.02 mL/min/1.73 m^2^, *p* < 0.0001, Table 2). After adjustments, a positive association was only detected in Lifelines (slope 0.17 ± 0.02 mL/min/1.73 m^2^, *p* < 0.0001, Table 2). We found no significant association between the protein diet score and eGFR in the Young Finns Study.

### 3.6. Association Total Protein E% and eGFR

Before adjustments, we found an inverse association in both NQplus (slope −0.74 ± 0.31 *E*%/mL/min/1.73 m^2^, *p* = 0.02, Table 2) and Lifelines (slope −0.60 ± 0.03 *E*%/mL/min/1.73 m^2^, *p* < 0.0001, Table 2), between the total protein intake (*E*%) and eGFR. For Lifelines, the association changed into a positive association after adjustments (slope 0.08 ± 0.02 *E*%/mL/min/1.73 m^2^, *p* < 0.0001, Table 2). However, in NQplus (slope −0.56 ± 0.25 *E*%/mL/min/1.73 m^2^, *p* = 0.02, Table 2), the inverse association persisted after further adjustments.

### 3.7. Association between Plant to Animal Protein Ratio and eGFR

Adjusted analyses showed a positive association between the plant to animal protein ratio and eGFR in both NQplus (slope: 3.96 ± 1.04 mL/min/1.73 m^2^, *p* < 0.001, Table 2) and Lifelines (slope: 1.14 ± 0.10 mL/min/1.73 m^2^, *p* < 0.001, Table 2). Unadjusted analyses also showed positive associations.

For the adjusted analyses, the variability of R-squared ranged from 0.00–0.23 for protein score versus. HbA1c and between 0.00–0.45 for protein score versus eGFR, respectively.

The relationship between the protein diet score and either HbA1c or eGFR was not qualitatively modified by age, gender, or BMI (Table 3).

## 4. Discussion

In the current cross-sectional study, we aimed (1) to develop a protein score capturing both relative quantity and source (plant versus animal) of dietary protein and (2) to examine the association between this score and markers of diabetes and renal function. We developed a protein score with a maximum range from 0 to 20 based on the FFQ data from the study populations investigated. In practice, the scores ranged between 8.0 and 12.0 across all three populations. The significant associations shown in Lifelines were partly reproduced by NQplus and the Young Finns Study, although in these smaller sample size studies, trends were more heterogeneous and affected by adjustment for potential confounders. In particular, the associations between the total protein score and both risk markers HbA1c and eGFR showed consistent patterns across all cohorts when compared to results for the separate components, i.e., animal and plant protein levels (Table S1). A higher total protein diet score was associated with a lower HbA1c and an increase in eGFR after adjustment for the potential confounders in Lifelines, but with no significant relationship in NQplus or the Young Finns Study.

Dietary protein has previously been shown to be beneficial, leading to better glycemic control in T2D [38]; hence, a higher protein diet has been advocated for glycemic control in individuals with T2D [39]. However, when evaluating the association between a higher protein score and HbA1c, it must also be taken into account that dietary protein will always substitute either carbohydrate and/or fat. In a recent study, even a relatively small variation in the proportion of fat and carbohydrates were significantly associated with metabolic risk factors in patients with T2D [40]. Although we adjusted for fat *E*%, residual confounding by other substances related to meat intake might be present. Previous studies have suggested several plausible mechanisms linking red and processed meat metabolites, including sodium, heme iron, saturated fatty acids, advanced glycation end products, nitrites and nitrates, to an increased risk of T2D [41,42,43]. In Lifelines, the protein score, protein intake and plant protein were associated with lower HbA1c. The optimal amount and quality of protein for prevention of T2D is still controversial [44]. In a recent study by Virtanen et al., high protein intake was not independently associated with risk of T2D, but the quality of protein was of importance, favouring plant over animal protein in the prevention of T2D [44]. In the study of Malik et al. [10], conducted in three ongoing prospective cohort studies: Nurses’ Health Study (NHS), Nurses’ Health Study II (NHS II) and Health Professionals Follow-up Study (HPFS)), a higher intake of total protein (*E*%) was positively associated with a higher risk of T2D, but this was shown to be largely due to animal protein intake. In contrast, intake of plant protein was associated with a lower risk of T2D. Results from the European Prospective Investigation into Cancer and Nutrition (EPIC)-NL study [45] also suggested a significantly increased diabetes risk over the quartiles of total protein intake after initial dietary adjustments. However, after further adjustment for waist circumference and BMI, the association was no longer significant. Similar results were observed for animal protein intake (*E*%), whereas vegetable protein intake was not related to T2D [45]. These results are consistent with the current findings observed in our dataset, which show an inverse association between plant protein intake and HbA1c. In the (EPIC)-InterAct case-cohort study [46], diabetes incidence was 17% higher in individuals with the highest protein intake, again largely explained by animal protein intake.

In contrast to these studies, we did not find the same association between total and animal protein and increased HbA1c values. This may be explained by differences in study population and the range of protein intakes. In the present analyses, median protein intake ranged from 0.95 to 1.3 g protein/kg body weight/day across the studies, which is within the daily recommended intake for adults [47]. Furthermore, variation in the protein diet score was low, reflected in the fact that 50% of the population had a range of 8.0 to 12.0. In addition, the difference in the findings may be due to differences in definitions of sources of animal protein or misclassification. The correlation between self-reported protein intake and urinary excretion of total nitrogen measured in the NQplus study was 48%.

Higher protein diet score and total protein intake was related to an increase in eGFR in Lifelines. In NQplus, we found an inverse association between total protein and eGFR. Based on data from the National Health and Nutrition Examination Survey (NHANES), Berryman et al. found no significant associations between total protein intake, or intake of animal or plant protein, and eGFR [48]. Similar results were found in a prospective cohort study by Halbesma et al. [49] and in the Nurses’ Health Study [50]. We suspect that differential residual confounding may be partly the cause of these discrepancies. In contrast, in a sub-study of the OmniHeart Trial, in a randomised three-period crossover feeding design, a protein-rich diet (48% plant-based) increased eGFR compared to diets rich in carbohydrates and unsaturated fat [51]. Similar to these findings, Frank et al. [13] showed a significant increase in eGFR with a higher protein diet in a randomised, crossover feeding study. According to Marckmann et al., an increase in eGFR is explained by an acute increase in renal plasma flow and eGFR due to a higher protein intake. The increase is maintained over weeks to months if protein intake is kept high. This condition, glomerular hyperfiltration, may have serious long-term effects on renal health [52]. In contrast, Bankir et al. stated that an increase in eGFR is likely to be a normal adaptation of the kidney to increased protein intake, and hence leads to higher urinary urea concentration [53], rather than being a reflection of poor renal function.

The strengths of the protein diet score developed in our study include easy calculations from FFQ data, as well as simple application and qualitative interpretation e.g., in term of risk prediction. However, a limitation in this protein score is that it is only applicable once population strata are generated. Limitations of FFQ data also apply to the protein score, and include potential confounding and measurement error [54]. Furthermore, it may be comparable across populations because of the use of percentile groups in a relative ranking, and adjustments for protein quality. The present study showed that the protein score was comparable across three study populations within northern Europe with a large number of participants. It can be argued that confounding is also controlled when protein quantity and source are considered simultaneously, in contrast to when the two elements of the score are analysed separately.

By nature, a weakness of the protein score is that it aggregates and condenses information, possibly capturing only some features of the dietary energy composition. Also, the score may not be directly applicable, i.e., quantitatively interpretable, in clinical practice because it is based on the above mentioned relative cut-off points. Our study also has other limitations. As with any cross-sectional investigations, conclusions regarding causality cannot be drawn and long-term intake patterns may not necessarily be captured. In addition, there are large differences in sample size between the trials, which may be responsible for the presence of significant effects in the Lifelines study, but the lack of such effects in the two other studies. However, the overall magnitude of the associations is very small, and thus unlikely to be of any clinical significance.

A weak point in all dietary analysis studies is the validity of self-reported dietary data, which is always debatable. The inherent limitations of over- and underreporting in self-reported dietary data must be acknowledged. In addition, absolute protein is often underreported with FFQ [31]. In the current study, we validated the FFQ used in NQplus with a urinary biomarker. It showed a reasonable agreement, with an attenuation factor of 0.48. This value is quite high when compared with a similar large study from Freedman et al. [31], which pooled five large US validation cohorts of dietary self-report instruments. They found an average attenuation factor for reported protein intake by FFQ of only 0.17.

## 5. Conclusions

In conclusion, we developed a protein diet score using cross-sectional data from three large European population studies. The protein score was comparable across these diverse study populations. We found some evidence that a higher protein score (higher intake of total protein and plant to animal protein) was associated with lower HbA1c values and with a higher eGFR. This study provides some evidence supporting the notion that both quantity and source of proteins (plant to animal protein ratio) are determining factors on their effect on HbA1c and eGFR. However, further studies are needed to clarify the usefulness of the novel protein score in long-term population and intervention studies, as well as in other health conditions.

## Figures and Tables

**Table 1:** Characteristics of the study populations (*n* =76,777).

Study	NQplus (*n* = 492)	Lifelines (*n* = 75,131)	Young Finns Study (*n* = 1154)
**Age**-years	53.0 (44.0; 60.0)	42.0 (32.0; 49.0)	39.0 (33.0; 42.0)
**Age** <44 years % (no.)	24.6 (121)	59.9 (44,992)	83.3 (961)
**Age** >44 years % (no.)	75.4 (371)	40.1 (30,139)	16.7 (193)
**Males**-% (no.)	61.0 (312)	38.8 (29,145)	59.7 (691)
**Age** <44 years (no.) (M/F)	(265/227)	(16,969/28,023)	(574/387)
**Age** >44 years (no.) (M/F)	(47/74)	(12,176/17,963)	(117/76)
**Education**-% (no.)			
- Low	0.4 (2)	1.7 (1253)	56.7 (655)
- Medium	42.3 (208)	63.9 (48,038)	22.95 (264)
- High	57.3 (282)	34.2 (25,729)	20.4 (235)
**Body mass index**-kg/m^2^	24.8 (22.7; 26.9)	24.7 (22.6; 27.3)	25.0 (22.4; 27.8)
**Physical activity**-MET–min/week	2376 (1380; 2950)	2205 (1260; 3582)	792 (180.0; 1899)
**Smoking status**-% (no.)			
- Never	54.7 (268)	50.3 (37,821)	53.0 (611)
- Former	37.4 (184)	27.8 (20,895)	23.8 (275)
- Current <10 cigarettes/day	3.4 (17)	10.5 (7881)	15.5 (179)
- Current ≥10 cigarettes/day	4.5 (22)	11.4 (8534)	7.7 (89)
**HbA1c** (mmol/mol)	35.5 (33.3; 37.0)	36 (34.0; 38.0)	36.0 (34.0; 38.0)
**eGFR** (mL/min/1.73 m^2^)	91.1 (81.7; 100.1)	100.0 (89.3; 109.5)	100.5 (90.2; 107.9)
**Energy intake**-calories/day	2039 (1728; 2456)	1983 (1639; 2386)	2217 (1834; 2653)
**Protein diet score**	10.0 (8.0; 12.0)	10.0 (8.00; 12.0)	10.0 (9.0; 12.0)
**Total protein intake**			
- Total protein-(*E*%/day)	14.6 (13.1; 15.9)	14.6 (13.3; 16.0)	17.4 (16.0; 19.0)
- Protein-(g/kg body weight/day)	0.99 (0.79; 1.17)	0.95 (0.78; 1.13)	1.3 (1.1; 1.6)
**Animal protein-**(*E*%/day)	7.7 (6.3; 9.3)	8.4 (7.1; 9.9)	12.3 (10.8; 14.0)
**Plant protein**-(*E*%/day)	6.6 (5.9; 7.4)	6.1 (5.5; 6.7)	3.8 (3.2; 4.3)
**Plant to animal protein ratio**	0.9 (0.7; 1.1)	0.72 (0.58; 0.90)	0.3 (0.2; 0.4)
**Red meat**-(g/day)	34.8 (19.2; 45.5)	38.6 (23.4; 50.5)	79.4 (55.5; 106.3)
**Processed meat**-(g/day)	16.9 (6.1; 31.1)	17.6 (8.4; 31.1)	38.1 (23.0; 64.5)
**Poultry**-(g/day)	9.5 (5.3; 15.0)	9.6 (6.1; 15.0)	26.0 (20.3; 60.8)
**Fish**-(g/day)	14.5 (8.2; 16.2)	10.5 (4.2; 16.4)	24.0 (16.6; 36.2)
**Eggs**-(g/day)	8.9 (7.1; 17.9)	7.2 (4.5; 17.9)	19.3 (13.9; 25.9)
**Dairy**-(g/day)	324.0 (211.8; 456.6)	295.7 (186.0; 426.1)	546.6 (343.4; 779.7)
**Cereals**-(g/day)	190.4 (140.3; 254.0)	181.2 (137.5; 234.0)	123.9 (91.8; 166.7)
**Legumes**-(g/day)	35.7 (19.2; 67.3)	4.4 (0.0; 13.3)	8.2 (4.7; 12.5)
**Total carbohydrate**			
- Total carbohydrate-(*E*%/day)	43.3 (39.7; 47.1)	45.2 (41.8; 48.7)	46.1 (42.4; 49.8)
- Total carbohydrate-( g/day)	220 (178.5; 269.2)	223.3 (182.0; 271.4)	254.7 (206.7; 309.2)
**Total fat**			
- Total fat-(*E*%/day)	35.6 (31.7; 39.2)	35.4 (32.1; 38.4)	32.6 (29.5; 35.6)
- Total fat-(g/day)	80.8 (62.9; 102.6)	77.6 (61.4; 96.4)	79.6 (64.4; 96.8)
**Alcohol consumption-**(g/day) % (no)			
- 0 g/day	3.9 (19)	2.5 (1875)	0.8 (9)
- >0–6 g/day	41.7 (205)	54.4 (40,775)	56.2 (646)
- 6–12 g/day	21.1 (104)	23.0 (17,243)	24.6 (283)
- >12 g/day	33.3 (164)	20.3 (15,238)	18.4 (212)
**Glycemic index**	53.1 (50.7; 55.4)	56.0 (54.1; 57.8)	51.0 (48.3; 53.8)
**Glycemic load**	117.4 (93.1; 146.7)	125.0 (100.8; 153.2)	129.8 (104.7; 159.9)

Characteristics are shown as median and IQR: interquartile range or as % (no.). eGFR: estimated glomerular filtration rate; *E*%: energy percentage; g: gram; HbA1c: glycated hemoglobin; low education: no education or primary education; medium education: lower or preparatory vocational education, lower general secondary education, intermediate vocational education or apprenticeship, higher general secondary education, or pre-university secondary education; high education: higher vocational education or university; F: females; M: males; MET: metabolic equivalent of task.

**Table 2:** Associations of glycated haemoglobin (HbA1c) and estimated glomerular filtration rate (eGFR) (estimate ± SE) across quintiles energy-adjusted protein diet score and quintiles of its energy-adjusted components.

Study	NQplus (*n* = 492)			Lifelines (*n* = 75,131) **			Young Finns Study (*n* = 1154)		
Variable	Slope ± SE	*p*-Value	*R* ^2^	Slope ± SE	*p*-Value	*R* ^2^	Slope ± SE	*p*-Value	*R* ^2^
**HbA1c**									
**Protein diet score**									
- Unadjusted	−0.04 ± 0.04	0.368	0.002	−0.0003 ± 0.01	0.95	0	0.01 ± 0.03	0.743	0
- Adjusted	−0.02 ± 0.04	0.722	0.229	−0.02 ± 0.01	<0.001	0.154	−0.02 ± 0.03	0.635	0.088
**Total protein (*E*%)**									
- Unadjusted	0.03 ± 0.06	0.671	0.0003	0.07 ± 0.01	<0.001	0.002	0.05 ± 0.04	0.211	0.001
- Adjusted	−0.04 ± 0.06	0.480	0.229	−0.03 ± 0.01	<0.001	0.154	0.0006 ± 0.02	0.988	0.088
**Plant to animal protein ratio**									
- Unadjusted	−0.49 ± 0.27	0.072	0.006	−0.19 ± 0.039	<0.001	0.001	−0.44 ± 0.66	0.505	0.0003
- Adjusted	−0.06 ± 0.26	0.823	0.229	−0.04 ± 0.03	0.142	0.154	−0.17 ± 0.71	0.810	0.089
**eGFR**									
**Protein diet score**									
- Unadjusted	0.53 ± 0.21	0.02	0.013	−0.08 ± 0.02	<0.0001	0.000	−0.16 ± 0.12	0.164	0.002
- Adjusted	0.32 ± 0.18	0.074	0.447	0.17 ± 0.02	<0.0001	0.391	0.08 ± 0.12	0.474	0.152
**Total protein (*E*%)**									
- Unadjusted	−0.74 ± 0.31	0.02	0.012	−0.601 ± 0.03	<0.0001	0.007	0.12 ± 0.15	0.445	0.0005
- Adjusted	−0.56 ± 0.25	0.02	0.449	0.08 ± 0.02	<0.0001	0.390	−0.02 ± 0.15	0.904	0.152
**Plant to animal protein ratio**									
- Unadjusted	6.46 ± 1.29	<0.0001	0.048	1.32 ± 0.09	<0.0001	0.003	−2.28 ± 2.52	0.367	0.0007
- Adjusted	3.96 ± 1.04	<0.001	0.460	1.14 ± 0.10	<0.001	0.391	2.17 ± −2.61	0.405	0.152

Change in HbA1c (mmol/mol) and eGFR (mL/min/1.73 m^2^) respectively, per 1 unit change in protein score. ** *n* = 69462 due to missing values of HbA1c model adjusted for age, gender, education (low/middle/high), alcohol (0 g/day, >0–6 g/day, 6–12 g/day, ≥12 g/day), smoking status (never, former, current <10 cigarettes/day, current ≥10 cigarettes/day), light intense, moderate intense, and intense physical activity (MET: minutes/week); total fat (*E*%), GI and BMI. Abbreviations: eGFR: estimated glomerular filtration rate, *E*%: energy percentage, HbA1c: glycated hemoglobin.

**Table 3:** Stratified analyses of the associations of HbA1c and eGFR (estimate ± SE).

Variable	NQPlus (*n* = 492)	Lifelines (*n* = 75,131)	Young Finns Study (*n* = 1154)
	Slope ± SE	Slope ± SE	Slope ± SE
**HbA1c**			
**Age**			
<44 years	−0.073 ± 0.104	0.149 ± 0.022 *	−0.013 ± 0.033
≥44 years	−0.250 ± 0.036 *	0.219 ± 0.024 *	0.016 ± 0.094
**Gender**			
Men	−0.012 ± 0.058	−0.016 ± 0.008 *	−0.008 ± 0.039
Women	0.0004 ± 0.072	−0.020 ± 0.006 *	0.005 ± 0.056
**BMI**			
<25 kg/m^2^	−0.009 ± 0.055	−0.012 ± 0.006	−0.039 ± 0.041
25–30 kg/m^2^	−0.021 ± 0.076	−0.033 ± 0.008 *	0.005 ± 0.048
≥30 kg/m^2^	−0.012 ± 0.044	0.005 ± 0.018	−0.012 ± 0.031
**eGFR**			
**Age**			
<44 years	0.040 ± 0.414	−0.019 ± 0.006 *	0.131 ± 0.129
≥44 years	0.424 ± 0.199	−0.012 ± 0.008	−0.053 ± 0.275
**Gender**			
Men	0.191 ± 0.277	0.189 ± 0.022 *	0.016 ± 0.165
Women	0.124 ± 0.319	0.137 ± 0.022 *	−0.098 ± 0.181
**BMI**			
<25 kg/m^2^	0.227 ± 0.234	0.164 ± 0.022 *	−0.014 ± 0.167
25–30 kg/m^2^	0.252 ± 0.299	0.179 ± 0.03 *	−0.024 ± 0.193
≥30 kg/m^2^	0.858 ± 0.822	0.117 ± 0.049 *	0.621 ± 0.333

Change in age (years), gender, and BMI (kg/m^2^) respectively, per 1 unit change in protein score. Model adjusted for age, gender (except for gender-stratified models), education (low/middle/high), alcohol (0 g/day, >0–6 g/day, 6–12 g/day, ≥12 g/day), smoking status (never, former, current <10 cigarettes/day, current ≥10 cigarettes/day), light intense, moderate intense, and intense physical activity (MET: minutes/week), Fat (en%), GI and BMI (kg/m^2^); * *p* < 0.05.

## Supplementary Materials

The following are available online at www.mdpi.com/2072-6643/9/7/763/s1, Table S1: Associations of HbA1c and eGFR (mean ± SE) across quintiles energy-adjusted protein diet score and quintiles of its energy-adjusted components.
