# Supplementary material for: A Protein Diet Score, Including Plant and Animal Protein, Investigating the Association with HbA1c and eGFR—The PREVIEW Project
Source: Nutrients. 2017 Jul 17;9(7):763. doi: 10.3390/nu9070763 (PMC5537877; doi:10.3390/nu9070763)
Supplement: Supplementary file 1 [file nutrients-09-00763-s001.docx]

**Table S1: Associations of HbA_1c_ and eGFR (estimate ± SE) energy-adjusted protein diet score and of its energy-adjusted components**

|  | | | | | | | | | |
| --- | --- | --- | --- | --- | --- | --- | --- | --- | --- |
| Study | **NQplus (*n* = 492)** | | | **Lifelines (*n* = 75,131) **** | | | **The Young Finns Study (*n* = 1154)** | | |
| Variable | **Slope ± SE** | ***p*-value** | **R^2^** | **Slope ± SE** | ***p*-value** | **R^2^** | **Slope ± SE** | ***p*-value** | **R^2^** |
| **HbA_1c_** | | | | | | | | | |
| **Animal protein (E%)** |  |  |  |  |  |  |  |  |  |
| - Unadjusted | 0.07 ± 0.06 | 0.257 | 0.003 | 0.074 ± 0.006 | <0.001 | 0.002 | 0.06 ± 0.04 | 0.104 | 0.002 |
| - Adjusted | −0.03 ± 0.06 | 0.593 | 0.229 | −0.011 ± 0.006 | 0.07 | 0.154 | 0.02 ± 0.04 | 0.548 | 0.088 |
| **Plant protein (E%)** |  |  |  |  |  |  |  |  |  |
| - Unadjusted | −0.15 ± 0.11 | 0.172 | 0.004 | −0.064 ± 0.014 | <0.001 | 0.000 | 0.02 ± 0.11 | 0.833 | 0.000 |
| - Adjusted | −0.03 ±0.11 | 0.808 | 0.229 | −0.049 ± 0.014 | <0.001 | 0.154 | −0.004 ± 0.11 | 0.967 | 0.089 |
|  | | | | | | | | | |
| **eGFR** | | | | | | | | | |
| **Animal protein (E%)** | | | | | | | | | |
| - Unadjusted | −1.29 ± 0.28 | <0.0001 | 0.041 | −0.551 ± 0.02 | <0.0001 | 0.007 | 0.21 ± 0.14 | 0.125 | 0.002 |
| - Adjusted | −0.86 ± 0.22 | <0.001 | 0.460 | −0.056 ± 0.02 | 0.008 | 0.390 | −0.04 ± 0.14 | 0.795 | 0.152 |
| **Plant protein (E%)** | | | | | | | | | |
| - Unadjusted | 2.17 ± 0.51 | <0.0001 | 0.035 | 0.221 ± 0.052 | <0.0001 | 0.000 | −0.54 ± 0.41 | 0.184 | 0.002 |
| - Adjusted | 1.48 ± 0.43 | <0.001 | 0.457 | 0.583 ± 0.046 | <0.0001 | 0.391 | 0.53 ± 0.42 | 0.203 | 0.153 |

Change in HbA1c (mmol/mol) and eGFR (ml/min/1.73 m^2^), respectively, per 1 unit change in protein score. ** *n* = 69462 due to missing values of HbA_1c._ Adjusted for age, gender, education (low/middle/high), , alcohol (0 g/d, >0-6 g/day, 6–12 g/day, ≥12 g/day), smoking status (never, former, current <10 cigarettes/day, current ≥ 10 cigarettes/day), light intense, moderate intense, and intense physical activity (MET-minutes/week); total fat (E%), GI and BMI. Abbreviations: eGFR: estimated glomerular filtration rate, E%: energy percentage, HbA_1c_: glycated hemoglobin.
